# Add-on-LUCAS2™-Reanimation am NEF Innsbruck

**DOI:** 10.1007/s00101-022-01112-z

**Published:** 2022-04-07

**Authors:** D. Schwaiger, A. Zanvettor, A. Neumayr, M. Baubin

**Affiliations:** Universitätsklinik für Anästhesie und Intensivmedizin, Medizinische Universität Innsbruck, Anichstraße 35, 6020 Innsbruck, Österreich

**Keywords:** Herz Kreislauf Stillstand, Mechanische CPR, Rettungsdienst, Prähospital, Überleben, Cardiac arrest, Mechanical CPR, EMS, Pre-hospital, Survival

## Abstract

**Studienziel:**

Ziele sind die Verlaufsanalyse und der Vergleich mit ausschließlich manuell reanimierten Patienten sowie die Erfassung der Einflussfaktoren bei Patienten, bei denen die mechanische Thoraxkompressionshilfe Lund University Cardiac Assist System (LUCAS2^TM^) als Add-on-Therapie am Notarzteinsatzfahrzeug (NEF) Innsbruck verwendet wurde.

**Material und Methodik:**

Retrospektive Verlaufsdatenanalyse von Patienten im Studienzeitraum 01.01.2014 bis 31.12.2019 des NEF Innsbruck aus dem Deutschen Reanimationsregister (GRR), bei denen LUCAS2™ nach notärztlicher Anordnung als Add-on-Therapie verwendet wurde.

**Ergebnis:**

Bei 653 Reanimationen kam es zu 123 Add-on-LUCAS2™-Anwendungen (18,8 %). Von allen Patienten überlebten 16,2 % die ersten 30 Tage. Mithilfe der Add-on-LUCAS2^TM^ Anwendung überlebten 7,3 % (9/123) aller Add-on-LUCAS2™-Reanimationen bzw. 1,4 % (*n* = 9) aller CPRs. Bei 8/9 Add-On-LUCAS2™-„30 Tage-Überlebenden“ war der Herz-Kreislauf-Stillstand (HKS) beobachtet, und eine Laien-CPR wurde durchgeführt. Als Primärrhythmus wiesen 8/9 Kammerflimmern auf.

Im Vergleich zur ausschließlich manuellen CPR wurde eine Add-on-LUCAS2™-Reanimation hoch signifikant (*p* < 0,001) häufiger bei jüngeren, bei männlichen Patienten, in der Öffentlichkeit, bei schockbarem Erstrhythmus und beim Transport eingesetzt sowie signifikant häufiger bei beobachteten HKS (*p* < 0,05).

Die 30-Tage-Mortalität bei additiver Lysetherapie betrug 100 %.

**Diskussion:**

Durch die Verwendung der Add-on-LUCAS2™-CPR kann eine prozentuelle Erhöhung der Überlebensrate erzielt werden und erscheint somit vorteilhaft (1,4 % in dieser Studie). Durch diese kann bei Patienten mit günstigen Prognosefaktoren eine hochwertige HDM auch bei technisch aufwendiger Bergung (Drehleiter, Stiegenhaus, Transport im RTW) durchgeführt und somit ein Transport ermöglicht werden. Jedoch kommt es dabei zu einer höheren Aufnahmerate unter CPR und somit zur Verlagerung der Therapiezielentscheidung in den Schockraum.

## Abstract (en)

**Study goal:**

The study goals were to analyze the course and compare it with patients who were only resuscitated manually as well as to record the influencing factors in patients in whom the mechanical chest compression aid LUCAS2™ was used as an add-on treatment at the NEF Innsbruck.

**Material and methodology:**

Retrospective history data analysis of patients in the study period from 01.01.2014 to 31.12.2019 of the NEF Innsbruck from the German Resuscitation Register (GRR), in which LUCAS2™ was used as an add-on treatment according to an emergency doctor’s order.

**Result:**

A total of 123 add-on LUCAS2™ applications (18.8%) were performed in 653 resuscitations. Of all patients 16.2% survived the first 30 days. By using add-on-LUCAS2^TM^ application 7.3% (9/123) of all add-on LUCAS2^TM^ resuscitations and 1.4% (*n* = 9) of all CPR survived. Cardiac arrest was observed in 8/9 add-on LUCAS2™ 30-day survivors and bystander CPR was performed and 8/9 showed ventricular fibrillation as the primary rhythm.

Compared to manual CPR alone, add-on LUCAS2™ resuscitation was used highly significantly (*p* < 0.001) more frequently in younger, male patients, in public, in shockable initial rhythms and during transport, and significantly more frequently in observed cardiac arrest (*p* < 0.05).

The 30-day mortality with additive lysis treatment was 100%.

**Discussion:**

By using add-on LUCAS2™ CPR a percentage increase in survival rate can be achieved and thus appears advantageous (1.4% in our study). This means that high-quality CPR can be carried out on patients with favorable prognostic factors, even with technically complex rescue operations (turntable ladder, staircase, transport in an ambulance) and thus transport can be made possible; however, there is a higher admission rate under CPR and thus the treatment target decision is shifted to the shock room.

## Hinführung zum Thema

In Österreich verstarben im Jahr 2020 91.599 Menschen, wobei 32.676 (35,7 %) Tode auf Herz-Kreislauf-Erkrankungen und deren Komplikationen zurückzuführen sind. Erkrankungen des Herz-Kreislauf-Systems führen die Todesursachenstatistik unverändert an, gefolgt von Krebserkrankungen [1]. Der präklinische Herz-Kreislauf-Stillstand (HKS) zählt zu den führenden Todesursachen in Europa [2]. 70 % davon sind auf die Pathologie einer koronaren Herzkrankheit zurückzuführen [3]. Die komplexe präklinische Behandlung des HKS gilt als Surrogatmarker für die präklinische Versorgung und ist mit einem hohen Aus- und Weiterbildungsaufwand verbunden.

## Hintergrund

Im Jahr 2019 wurden von den 13 Tiroler bodengebundenen Notarzteinsatzfahrzeugen (NEF) 482 Reanimationsversuche durchgeführt, davon 113 (23,5 %) durch das Team des NEF Innsbruck [4].

Die frühzeitige und hochwertige Herzdruckmassage (HDM) erhöht die Chance auf die Rückkehr eines Spontankreislaufs (ROSC) signifikant [5–10], und mit ihr kann ein Herzminutenvolumen bis zu 20–30 % der Norm erreicht werden [11–13].

Die Verwendung einer mechanischen Thoraxkompressionshilfe ermöglicht das Freispielen menschlicher Ressourcen; negative Einflussfaktoren auf die HDM-Qualität wie Ermüdung können minimiert werden, Frequenz und Kompressionstiefe bleiben auch unter Transportbedingungen konstant.

Die mechanische Thoraxkompressionshilfe LUCAS2™ (Lund University Cardiac Assist System; Jolife AB, a part of Stryker, Lund, Sweden) gilt als Weiterentwicklung der Active-Compression-Dekompression(ACD)-Reanimation, bei der im Unterschied zur ACD-Reanimation die Thoraxdekompression nur bis zum Ausgangspunkt zum Zeitpunkt der Anlegung des Device stattfindet [14]. Trotz zahlreicher randomisierter LUCAS2™-Multizenterstudien konnte in keiner ein signifikanter Überlebensvorteil gegenüber einer ausschließlich manuellen CPR nachgewiesen werden [15–17].

Seit April 2011 kann LUCAS2™ im Ballungsraum Innsbruck vom jeweiligen Notarzt zur Add-on-Therapie vom Einsatzleiter Rettungsdienst angefordert werden.

## Methode

Innerhalb des Studienzeitraums vom 01.01.2014 bis 31.12.2019 wurden alle Add-on-LUCAS2™-Reanimationen (AoLCPR) und deren innerklinische Weiterversorgungsdaten aus dem Krankenhausinformationssystem der Universitätsklinik Innsbruck und dem Datensatz des Deutschen Reanimationsregisters (GRR) [18] erfasst, nachkontrolliert und statistisch analysiert.

Beim Parameter Einsatzort wurden die Kategorien Straße, Öffentlichkeit, Arztpraxis, Massenveranstaltung, Arbeitsplatz und Sportstätte als „öffentlich“ zusammengefasst.

**Figure 1:**
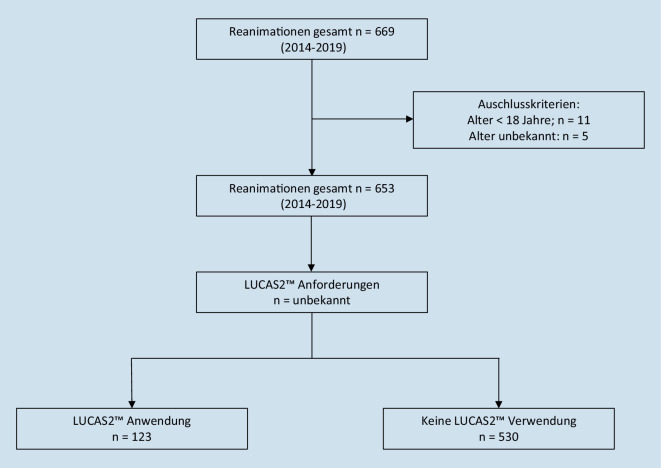

Ausgeschlossen wurden Patienten < 18 Jahre und/oder Patienten, bei denen kein Alter angegeben wurde (Abb. 1).

Ziele dieser Studie sind die Bestimmung des 30-Tage-Überlebens bei Patienten mit HKS nach Hinzuziehen von LUCAS2™ und die Erfassung der jeweiligen Einflussfaktoren am NEF Innsbruck. Die Outcomes dieser AoLCPR werden mit den ausschließlich manuellen Reanimationen (amCPR) desselben Zeitraums verglichen.

Erwartet wird, dass LUCAS2™ gehäuft bei HKS mit vermutlich guter Prognose (junges Alter, beobachteter Herz-Kreislauf-Stillstand, durchgeführte Ersthelferreanimation, schockbarer Erstrhythmus) und vorwiegend für den Transport der Patienten angefordert und verwendet wird.

### Statistische Analyse

IBM SPSS Statistics 26 (IBM Corporation, Armonk, NY, USA) wurde zur statistischen Auswertung verwendet. Baseline-Charakteristika wurden auf Signifikanz mittels Fisher-Exakt-Test, Mann-Whitney-U-Test und Chi-Quadrat untersucht. Ein zweiseitiger *p*-Wert < 0,05 wurde als statistisch signifikant bzw. < 0,001 als hochsignifikant eingestuft.

## Ergebnisse

### Allgemeine Ergebnisse

Im 6‑Jahres-Zeitraum wurde bei 18,8 % der reanimierten Patienten eine Add-on-LUCAS2™- CPR (AoLCPR) durchgeführt. Die durchschnittliche Anforderung betrug 20,5/Jahr.

Das Patientenalter unterscheidet sich in den Gruppen AoLCPR und „ausschließlich manuelle“ CPR (amCPR) signifikant. 78,9 % der AoLCPR- vs. 60,2 % der amCPR-Patienten waren männlich.

48,8 % der AoLCPR und 17,9 % der amCPR fanden in der Öffentlichkeit statt, wohingegen in einer Wohnung häufiger ausschließlich manuell reanimiert wurde (63,4 %). Bei 67,5 % der AoLCPR und bei 55,1 % der amCPR war der HKS beobachtet. Als erstdokumentierter EKG-Rhythmus wurde hoch signifikant häufiger Kammerflimmern bei AoLCPR (35,0 % vs. 19,2 %) und Asystolie bei amCPR (50,9 % vs. 26,8 %) dokumentiert (Tab. 1).

**Table 1:** 

		Add-on-LUCAS2™		Ausschließlich manuell		*p*-Wert	
		*n* = 123	%	*n* = 530	%		
Einsatzort	Öffentlicher Raum	60	48,8	95	17,9	< 0,001	< 0,001
	Wohnung	56	45,5	336	63,4	< 0,001	
	Sonstiger	2	1,6	16	3,0	n.s	
	Altenheim	0	0	69	13,0	< 0,001	
	Krankenhaus	1	0,8	4	0,8	n.s	
	Nicht dokumentiert	1	0,8	2	0,4	n.s	
	Nicht bekannt	3	2,4	8	1,5	n.s	
Beobachtet	Ja	83	67,5	292	55,1	< 0,05	< 0,05
Laien-CPR	Ja	65	52,8	296	55,8	n.s	n.s
Vermutete Ursache	Kardial	75	61,0	308	58,1	n.s	n.s
	Trauma	12	9,8	21	4,0	< 0,05	
	Hypoxie	9	7,3	60	11,3	n.s	
	Intoxikation	5	4,1	23	4,3	n.s	
	Verbluten	3	2,4	7	1,3	n.s	
	Ertrinken	1	0,8	0	0	n.s	
	ICB/SAB	1	0,8	5	0,9	n.s	
	Sepsis	0	0	1	0,2	n.s	
	Stroke	0	0	3	0,6	n.s	
	Metabolisch	0	0	6	1,1	< 0,05	
	Sonstiges	5	4,1	18	3,4	n.s	
	Nicht bekannt	12	9,8	78	14,7	n.s	
Erstrhythmus	VF/VT	43	35,0	102	19,2	< 0,001	< 0,001
	PEA	47	38,2	158	29,8	n.s	
	Asystolie	33	26,8	270	50,9	< 0,001	
∑ Schocks (Anzahl, wenn defibrilliert)	1 Schock	11	18,6	52	38,5	n.s	< 0,001
	Bis 3 Schocks	12	20,3	41	30,4	n.s	
	4–6 Schocks	20	33,9	32	23,7	< 0,05	
	7–9 Schocks	6	10,2	8	5,9	n.s	
	Mehr als 9 Schocks	10	16,9	2	1,5	< 0,05	
Lysetherapie	Während HKS	15	12,2	7	1,3	< 0,001	–
	Nach ROSC	1	0,8	3	0,6	n.s	
	Keine	107	87,0	520	98,1	< 0,05	
Aufnahme	Mit ROSC	12	9,8	222	41,9	< 0,001	< 0,001
	Unter laufender CPR	108	87,8	20	3,8	< 0,001	
	Keine Aufnahme	3	2,4	288	54,3	< 0,001	
Transportzeit (Median, SD)		5 (±5,09)	–	5 (±4,54)	–	n.s	n.s

*n* Fallzahl, *n.s* nicht signifikant, *SD* Standardabweichung

Eine Lysetherapie mit Tenecteplase während der CPR wurde bei AoLCPR hoch signifikant häufiger durchgeführt (12,2 % vs. 1,3 %).

2,4 % der AoLCPR- bzw. 54,3 % der amCPR-Patienten verstarben am Einsatzort (Tab. 1).

Bei allen LUCAS2™-Anwendungen wurde eine endotracheale Intubation durchgeführt. Der Beatmungsmodus (manuell, maschinell; 30:2 oder kontinuierliche Thoraxkompressionen) wurde nicht dokumentiert.

### Transport ins Zielkrankenhaus

97,6 % der AoLCPR- und 45,7 % der amCPR-Patienten wurden im internistischen oder traumatologischen Schockraum des Landeskrankenhaus Innsbruck aufgenommen, davon 9,8 % der AoLCPR- und 41,9 % der amCPR-Patienten mit anhaltendem ROSC.

16,5 % (*n* = 108) aller reanimierten Patienten wurden unter laufender AoLCPR und 3,1 % (*n* = 20) unter amCPR transportiert und aufgenommen. Von diesen 20 amCPR-Patienten verfiel bei 9 Patienten mit primärem ROSC der Kreislauf wieder während des Transports, und eine CPR wurde wieder begonnen; bei einem erfolgte eine Lysetherapie; bei einem konnte habitusbedingt keine AoLCPR durchgeführt werden. Weitere 9 Patienten wurden unter laufender amCPR eingeliefert. Weshalb bei diesen kein LUCAS2™ zu Anwendung kam, wurde nicht dokumentiert.

### Klinische Weiterversorgung, Outcome

55,4 % (*n* = 362) der reanimierten Patienten wurden in einen internistischen oder traumatologischen Schockraum aufgenommen.

Bei 9,2 % der aufgenommenen AoLCPR- und bei 24,4 % der aufgenommenen amCPR-Patienten wurde eine Koronarangiographie durchgeführt (*p* < 0,05) und bei 9,2 % der aufgenommenen AoLCPR und bei 27,3 % der aufgenommenen amCPR eine therapeutische Hypothermie eingeleitet (*p* < 0,001).

28,6 % aller reanimierten Patienten überlebten die ersten 24 h, davon wurde bei 3,7 % (*n* = 24) eine AoLCPR und bei 25,0 % (*n* = 163) eine amCPR durchgeführt (Tab. 2).

**Table 2:** 

Krankenhausaufnahmen		Add-on-LUCAS2™		Ausschließlich manuell		*p*-Wert	
		*n* = 120		*n* = 242			
Koronarangiographie	Ja	11	9,2	59	24,4	< 0,001	*p* < 0,05
	Nein	90	75,0	148	61,2	< 0,05	
	Nicht bekannt	19	15,8	35	14,5	n.s	
Therapeutische Hypothermie	Ja	11	9,2	66	27,3	< 0,001	*p* < 0,001
	Nein	82	68,3	113	46,7	< 0,001	
	Nicht bekannt	27	22,5	63	26,0	n.s	
24 h Überleben	Ja	24	19,7	163	67,4	< 0,001	–
30-Tage-Überleben	Ja	9	7,4	97	40,1	< 0,001	–

*n* Fallzahl, *SD* Standardabweichung

Von allen Patienten überlebten 16,2 % die ersten 30 Tage: 1,4 % (*n* = 9) der AoLCPR und 14,9 % (*n* = 97) der amCPR (einer während des Transports).

Ein zerebraler-Performance-Score (CPC) von 1–2 wurde bei 77,7 % (*n* = 7/9) bzw. 74,2 % (*n* = 72/97) der 30-Tage-Überlebenden nach AoLCPR bzw. nach amCPR festgestellt (Tab. 2).

AoLCPR-Patienten waren im Median 7 Jahre jünger als amCPR-Patienten. 78,9 % der AoLCPR- vs. 60,2 % der amCPR-Patienten waren männlich. 48,8 % der AoLCPR vs. 17,9 % der amCPR fanden in der Öffentlichkeit statt. 67,5 % der AoLCPR vs. 55,1 % der amCPR waren beobachtet und 35,0 % der AoLCPR wiesen VF/VT als Erstrhythmus auf.

Die 30-Tage-Mortalität bei additiver Lysetherapie betrug in beiden Gruppen 100 % (Tab. 3).

**Table 3:** 

		Add-on-LUCAS2™		Ausschließlich manuell	
		*n* = 9	%	*n* = 97	%
Alter (Median, SD)		75,00 (±15,33)		59 (±16,95)	
		–		–	
Geschlecht	Männlich	6	66,7	68	70,1
Einsatzort	Öffentlicher Raum	8	88,9	43	44,3
	Wohnung	1	11,1	45	46,4
	Sonstiger	0	0	0	0
	Altenheim	0	0	4	4,1
	Krankenhaus	0	0	2	2,1
	Nicht dokumentiert	0	0	1	1
	Nicht bekannt	0	0	2	2,1
Beobachtet	Ja	8	88,9	79	81,4
Laien-CPR	Ja	9	100	41	42,3
Vermutete Ursache	Kardial	8	88,9	63	64,9
	Intoxikation	0	0	10	10,3
	Hypoxie	0	0	8	8,2
	Trauma	0	0	2	2,1
	ICB/SAB	0	0	2	2,1
	Stroke	0	0	1	1
	Metabolisch	0	0	1	1
	Nicht bekannt	1	11,1	10	10,3
Erstrhythmus	Kammerflimmern	8	88,9	54	55,7
	PEA	1	11,1	37	38,1
	Asystolie	0	0,0	6	6,2
∑ Schocks (Anteil, wenn defibrilliert)	1 Schock	0	0	20	37,7
	Bis 3 Schocks	1	12,5	17	32,1
	4–6 Schocks	6	75	13	24,5
	7–9 Schocks	1	12,5	3	5,7
KH-Aufnahme	Unter laufender CPR	5	56,6	1	1
CPC	Gute zerebrale Leistungsfähigkeit	4	44,4	59	60,8
	Mäßige zerebrale Behinderung	3	33,3	13	13,4
	Schwere zerebrale Behinderung	0	0	4	4,1
	Koma, vegetativer Zustand	0	0	2	2,1
	Nicht bekannt	2	22,2	19	19,6
Transportzeit (Median, SD)		2,00	2,15	5	4,12

*n* Fallzahl, *SD* Standardabweichung

**Figure 2:**
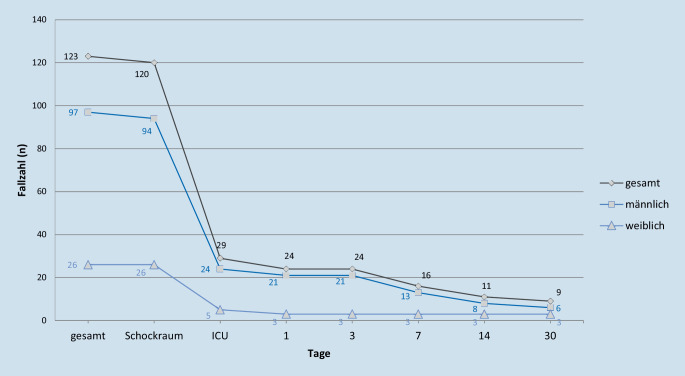

Von 120 transportierten Patienten wurden 29 auf eine Intensivstation aufgenommen, 24 überlebten die ersten 24 h. Ein geschlechtsspezifischer Unterschied bei der Aufnahme auf eine Intensivstation (24,7 % vs. 19,2 %) lässt sich nicht darstellen, jedoch überlebten die ersten 30 Tage prozentuell mehr Frauen eine Add-On-LUCAS2™-Reanimation als Männer (11,5 % (*n* = 6) vs. 6,2 % (*n* = 3)) (Abb. 2).

Die 30-Tage-Überlebenden einer AoLCPR wurden häufiger in der Öffentlichkeit reanimiert, hatten vermutlich eine kardiale Ursache und wiesen in 8/9 Fällen VF/VT als ersten EKG-Rhythmus auf (Tab. 3). Im Median waren die 30-Tage Überlebenden einer amCPR 16 Jahre jünger als die Überlebenden der AoLCPR.

## Diskussion

Unseres Wissens handelt es sich hierbei um die erste präklinische Outcome-Studie zu einer mechanischen Thoraxkompressionshilfe, bei welcher diese notärztlich indiziert angefordert, herangebracht und als Add-on-Therapie verwendet wird.

LUCAS2™ wurde hochsignifikant häufiger bei jüngeren Patienten, in der Öffentlichkeit, bei Kammerflimmern als Erstrhythmus und signifikant häufiger bei beobachtetem HKS angefordert und angewendet.

Dieses Ergebnis deckt sich mit der 2019 durchgeführten multizentrischen Registeranalyse des Deutschen Reanimationsregisters zwischen mechanischer und manueller CPR [19], wobei diese Studie als Endziel den „ROSC nach HKS“ hatte, während unsere Studie monozentrisch und bis zum 30-Tage-Überleben analysiert.

Unsere Ergebnisse zeigen, dass 9 (1,4 %) aller 106 30-Tage-Überlebenden (16,2 %) innerhalb des 6‑jährigen Studienzeitraums am NEF Innsbruck mithilfe einer AoLCPR überlebten. Fünf dieser 30-Tage-Überlebenden wurden unter laufender AoLCPR ins Krankenhaus eingeliefert. Dies sind 4,7 % der Überlebenden am NEF Innsbruck. Das Auftreten eines ROSC bei primärer Weiterführung einer amCPR ist für diese Patienten nicht auszuschließen. Vier der 30-Tage-Überlebenden einer AoLCPR konnten mit ROSC transportiert werden. Kein Patient überlebte eine additive Lysetherapie, weshalb die Kombination Lysetherapie und LUCAS2™-CPR seit Dezember 2018 am NEF Innsbruck nicht mehr empfohlen wird.

Wie in Abb. 2 ersichtlich, wurde durch das LUCAS2™-Add-On-Konzept der Todeszeitpunkt vielfach in die Klinik verlagert. Von insgesamt 120 transportierten Patienten mit einer AoLCPR verstarben 75,9 % im Schockraum. Bei keinem Patienten wurde eine extrakorporale Membranoxygenierung (ECMO) im Schockraum durchgeführt. Präklinisch stehen innerklinische Möglichkeiten wie Echokardiographie, Blutgasanalyse sowie eine Teamentscheidung unter mehreren Ärzten nicht zur Verfügung. Aufgrund dessen, der häufigeren CPR in der Öffentlichkeit sowie kurzer Transportwege ins Schwerpunktkrankenhaus könnte häufig ein Transport unter laufender CPR angestrebt worden sein.

Ein Patient der amCPR-Gruppe überlebte die CPR unter Transportbedingungen. Bei diesem Patienten verfiel – nach primären ROSC am Einsatzort – der Kreislauf während der Fahrt, und die Reanimation wurde wieder manuell begonnen. Somit kam LUCAS2™ aus logistischen Gründen hier nicht zum Einsatz.

In der klinischen Weiterversorgung unterscheiden sich die beiden Gruppen signifikant. Eine Koronarangiographie sowie eine therapeutische Hyperthermie wurden signifikant häufiger in der amCPR-Gruppe angewendet. Dies könnte aber auf deutlich erhöhte ROSC-Rate (41,9 % vs. 9,8 %) bei Aufnahme der amCPR-Patienten zurückzuführen sein.

Nachdem die Überlebensrate aus dem Patientengut des NEF Innsbruck über mehrere Jahre hinweg überdurchschnittlich gute Outcome-Daten zeigte (16,2 % 30-Tage-Überlebende im Studienzeitraum), ist ein Zuwachs dieser Rate nur mit hohem Aufwand erreichbar, bzw. bewirkt eine additive Therapie- oder Prozess-Option nur geringe Zuwächse.

Die internationalen LUCAS2™-Studien „the MECCA study report“ [15], „The Linc Randomized Trial“ [17] und „The PARAMEDIC Study“ [16] wurden zum Vergleich herangezogen (Tab. 4).

**Table 4:** 

	Add-On-LUCAS2™	PARAMEDIC Study	MECCA Study	LINC Trial
Design	„Retrospective monocenter study“	„Prospective multicenter study“	„Prospective multicenter study“	„Prospective multicenter study“
LUCAS™	Nach Anforderung	Fixer Bestandteil	Fixer Bestandteil	Fixer Bestandteil
Ausschlusskriterien	< 18 Jahre	< 18 Jahre, Trauma	< 21 Jahre, Trauma	< 18 Jahre, Trauma
Fallzahl (*n*)	132	1652	302	1300
30-Tage-Überleben	7,3 %	6 %	4 %	8,1 %
CPC 1‑2	77,7 %	74 %	Keine Angabe	93,8 %
Gesamtüberleben inkl. manuelle CPR	16,2 %	10,5 %	3,3 %	7,7 %

*n* Fallzahl, *SD* Standardabweichung

All diese sind Cluster-kontrollierte, multizentrische, randomisierte prospektive Paramedic-Studien, bei denen LUCAS2™ ein fixer Bestandteil der Ausrüstung war, und es wurden in diesen – im Gegensatz zu unserer Studie – ein traumatischer HKS und Schwangere ausgeschlossen.

Beim MECCA Study Report betrug das 30-Tage-Überleben in der LUCAS2™-Gruppe 4 %, in der manuellen Gruppe 3 %. Das neurologische Outcome wurde nicht angegeben [15].

Im LINC Trial überlebten 8,1 % der LUCAS2™- und 7,3 % der manuell reanimierten Patienten die ersten 30 Tage. Ein CPC von 1 bzw. 2 wurde bei 93,8 % der LUCAS2™-und bei 86,2 % der manuell reanimierten Patienten angegeben [17].

In der PARAMEDIC-Studie überlebten 6 % der mit LUCAS2™ und 7 % der ausschließlich manuell reanimierten Patienten die ersten 30 Tage. Bei 74 % der mit LUCAS2™ und bei 87 % der ausschließlich manuell reanimierten Patienten wurde ein CPC1 oder CPC2 angegeben [16].

Demgegenüber überlebten 16,2 % der reanimierten Patienten des NEF Innsbruck die ersten 30 Tage, davon 14,9 % nach amCPR und weitere 1,4 % nach zusätzlicher AoLCPR. 77,7 % bzw. 74,2 % der 30-Tage-Überlebenden nach einer AoLCPR bzw. amCPR wiesen einen CPC 1–2 auf. Hier ist zu berücksichtigen, dass eine AoLCPR häufig bei prolongierten Reanimationen und unter besonderen Umständen verwendet wird. Dies kann auch den Altersunterschied zwischen AoLCPR und amCPR erklären. Bei Einsätzen in der Öffentlichkeit – 8/9 der AoL-30-Tage-Überlebenden – wird der Einsatzleiter, der den LUCAS2™ in seinem Rettungsmittel mitbringt, oft zeitgleich mit dem NEF alarmiert und trifft u. U. vor oder zeitgleich ein, sodass eine eigene Nachforderung nicht notwendig ist. Diese Besonderheit wurde in der Auswertung nicht berücksichtigt.

Aufgrund dieser Unterschiede, besonders im Studiendesign, aber auch im deutlich höheren 30-Tage-Überleben an unserem Notarztsystem, ist ein Vergleich unserer Daten mit den genannten internationalen Multizenterstudien in vielerlei Hinsicht nicht sinnvoll.

Zusammenfassend lässt sich durch die additive AoLCPR-Strategie kein signifikant besseres Behandlungsergebnis im Vergleich zur ausschließlichen amCPR darstellen. Andererseits profitieren laut unserer Studie 9/653 (1,4 %) aller CPR-Patienten im Sinne eines 30-Tage-Überlebens von der AoLCPR-Strategie, das sind 9/123 (7,3 %) der 30-Tage-Überlebenden.

Durch eine AoLCPR kann bei Patienten mit günstigen Prognosefaktoren eine hochwertige HDM auch bei technisch aufwendiger Bergung (Drehleiter, Stiegenhaus) durchgeführt und somit ein Transport ermöglicht werden. Als Nebeneffekt kommt es jedoch zu einer höheren Aufnahmerate und somit zur Verlagerung der Therapiezielentscheidung in den Schockraum. Somit hat LUCAS2™ für uns weiterhin seine Berechtigung im präklinischen Setting auch in der dargestellten Add-on-Variante.

## Limitationen

Diese Arbeit unterliegt einer Reihe von Limitationen.

Die Patientenzahl im Studienzeitraum ist bezogen auf die hohe Prozessvariabilität des Reanimationsablaufes gering. So ergibt sich, dass diese Studie lediglich mit einem NEF-System bzw. einem LUCAS2™ sowie nur einem Weiterversorgungszentrum (Universitätsklinik Innsbruck) durchgeführt wurde. Multizentrische Studien rekrutieren mehr Daten, beinhalten jedoch heterogene Faktoren wie unterschiedliches Schockraummanagement, innerklinische Weiterversorgung und Therapiemöglichkeiten.

Ein Zeitangabe bezüglich der Dauer der Add-on-LUCAS2™-Therapie sowie eine Angabe des CPR-Modus (kontinuierlich, 30:2) wurden nicht dokumentiert.

Die am Einsatzort anwesenden Rettungskräfte benutzen LUCAS2™ im Einsatzfall und selten und sind im LUCAS2™-Realablauf nicht immer erfahren. Somit können sich längere Hands-off-Zeiten ergeben, was aber unbedingt vermieden werden soll.

## Fazit

Auf der Basis dieser retrospektiven Datenanalyse kann folgende Empfehlung für den Einsatz von LUCAS2™ ausgesprochen werden. LUCAS2™, wie auch andere mechanische Thoraxkompressionshilfen, sollen weiterhin bei klarer Indikation, wie bei nichtpraktikabler hochwertiger manueller CPR z. B. unter dem Transport, zum und im RTW, bei der Drehleiterbergung oder wenn diese ein Risiko für die Sicherheit der Rettungskräfte darstellt, verwendet werden. So wird dies auch in den ERC-Leitlinien empfohlen [20]. Die Entscheidung zur Verwendung von LUCAS2™ soll durch den Notarzt oder die Notärztin bzw. den Teamleiter unter genauer Abwägung der frühzeitigen Prognosefaktoren, wie beobachteter Herz-Kreislauf-Stillstand, suffiziente Ersthelfer-CPR und Erstrhythmus getroffen werden. Die Verwendung von LUCAS2™ in der Kombination mit einer Lysetherapie wird von uns nicht empfohlen.
